# Supplementary material for: Looking for ESKAPE Bacteria: Occurrence and Phenotypic Antimicrobial Resistance Profiles in Wild Birds from Northern and Central Italy Sites
Source: Antibiotics (Basel). 2025 Oct 14;14(10):1025. doi: 10.3390/antibiotics14101025 (PMC12561032; doi:10.3390/antibiotics14101025)
Supplement: Supplementary file 1 [file antibiotics-14-01025-s001.zip › antibiotics-3876574-supplementary.pdf]

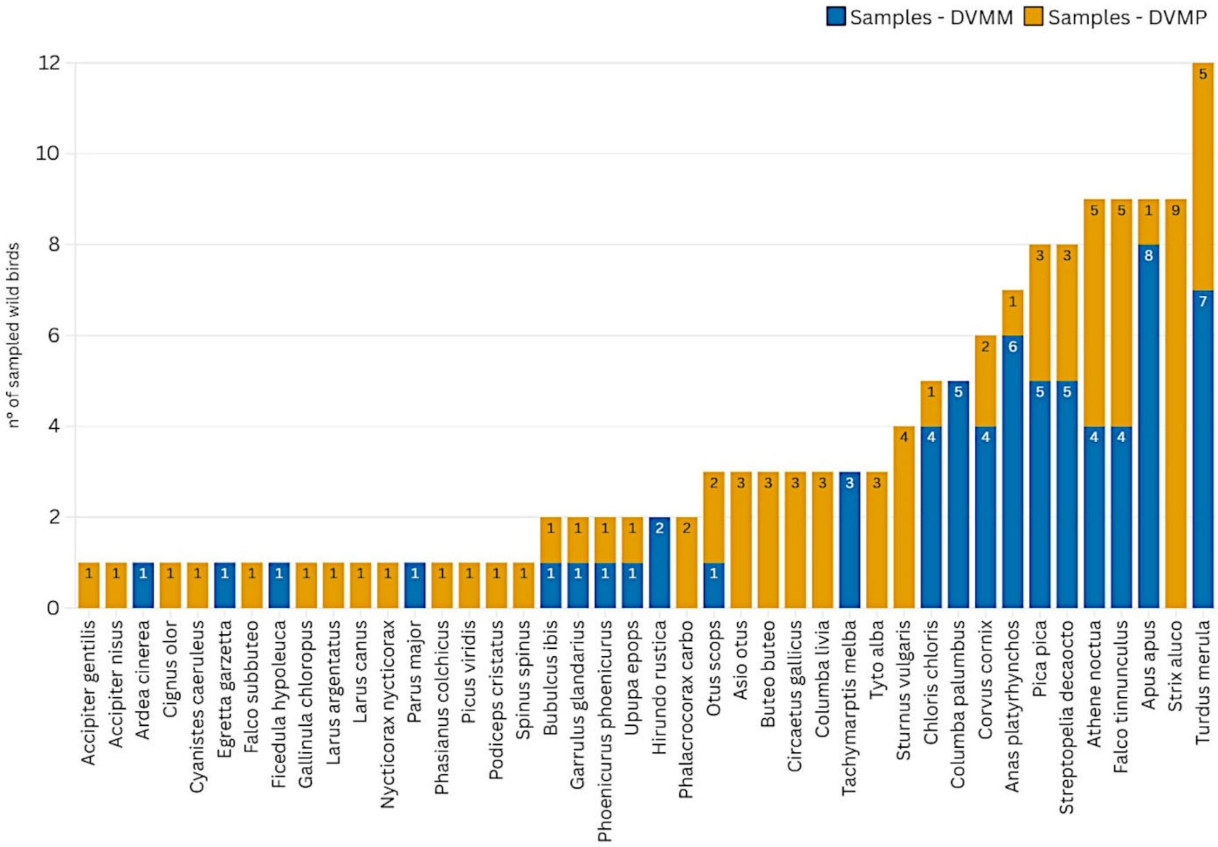

Figure S1. Distribution of sampled wild bird species (n = 141) across DVMM (n = 66) and DVMP (n = 75). Each bar represents the number of individuals per species, color-coded by sampling site (blue: DVMM; orange: DVMP), as reported in the legend.

Table S1. ESKAPE and other bacterial species isolated in birds from DVMM. Wild bird species are reported in rows with the sampling number, while bacterial species are reported in columns with the number of isolated strains.

	Isolated bacteria																										
**Wild bird species**	*A. baumannii* (n=1)	*C. braakii* (n=1)	*C. freundii* (n=2)	*E. hormaechei* (n=2)	*E. roggenkampii* (n=1)	*E. coli* (n=38)	*H. alvei* (n=2)	*K. aerogenes* (n=1)	*K. oxytoca* (n=1)	*K. pneumoniae* (n=8)	*K. gibsonii* (n=1)	*M. morganii* (n=3)	*P. hauseri* (n=1)	*P. mirabilis* (n=24)	*P. vulneris* (n=1)	*P. aeruginosa* (n=5)	*S. epidermidis* (n=1)	*S. saprophyticus* (n=1)	*S. simulans* (n=1)	*V. fluvialis* (n=2)	*V. teuberi* (n=1)	*E. avium* (n=1)	*E. casseliflavus* (n=1)	*E. faecalis* (n=33)	*E. faecium* (n=9)	*E. hirae* (n=3)	*E. thailandicus* (n=1)
Grey Heron (*Ardea cinerea*) (n=1)						1								1						1							
Cattle Egret (*Bubulcus ibis*) (n=1)						1								1						1							
Eurasian Scops-owl (*Otus scops*) (n=1)						1								1										1			
European Pied Flycatcher (*Ficedula hypoleuca*) (n=1)						1				1																	
Great Tit (*Parus major*) (n=1)																1											1
Little Owl (*Athene noctua*) (n=4)						3	1							3										2			
Common Redstart (*Phoenicurus phoenicurus*) (n=1)						1				1				1													
Common Woodpigeon (*Columba palumbus*) (n=5)						3								1										2	3		
Hooded Crow (*Corvus cornix*) (n=4)						2								4										4			
Little Egret (*Egretta garzetta*) (n=1)				1														1						1			
Eurasian Magpie (*Pica pica*) (n=5)	1	1				2			1			1		1							1			3			
Mallard (*Anas platyrhynchos*) (n=6)						3								1										4		2	
Common Kestrel (*Falco tinnunculus*) (n=4)						2								1					1					3			

Eurasian Jay<br>(*Garrulus glandarius*) (n=1)				1																			1	
Common Blackbird<br>(*Turdus merula*) (n=7)				6		1		2		1	1	4		1								3		1
Barn Swallow<br>(*Hirundo rustica*) (n=2)				1	1									1								1		
Alpine Swift<br>(*Tachymarptis melba*) (n=3)				1				2				1										1	1	
Common Swift<br>(*Apus apus*) (n=8)	1			4				1				3								1		6	2	
Eurasian Collared-dove<br>(*Streptopelia decaocto*) (n=5)		1		2				1	1			1	1	1							1	1	1	
Eurasian Hoopoe<br>(*Upupa epops*) (n=1)				1						1													1	
European Greenfinch<br>(*Chloris chloris*) (n=4)	1		1	2										1	1							1		

Table S2. ESKAPE and other bacterial species isolated in birds from DVMP. Wild bird species are reported in rows with the sampling number, while bacterial species are reported in columns with the number of isolated strains.

	Isolated bacteria																					
**Wild bird species**	*B. cereus* (n=1)	*C. koseri* (n=1)	*C. pseudotuberculosis* (n=1)	*E. coli* (n=46)	*E. hormaechei* (n=4)	*E. marmotae* (n=6)	*K. aerogenes* (n=1)	*K. oxytoca* (n=1)	*K. pneumoniae* (n=1)	*L. garviae* (n=1)	*L. adecarboxylata* (n=1)	*P. aeruginosa* (n=3)	*R. ornithinolytica* (n=1)	*R. planticola* (n=2)	*R. endophytica* (n=1)	*S.* Typhimurium (n=2)	*S. aureus* (n=2)	*S. saprophyticus* (n=1)	*S. xylosus* (n=4)	*E. faecium* (n=1)	*E. faecalis* (n=1)	*M. sciuri* (n=11)
Common Blackbird (*Turdus merula*) (n=5)	1			2		3											1					
Common Moorhen (*Gallinula chloropus*) (n=1)				1												1						
European Starling (*Sturnus vulgaris*) (n=4)				2	1	1				1									1			
Common Swift (*Apus apus*) (n=1)									1													
Blue Tit (*Cyanistes caeruleus*) (n=1)																1		1				
Eurasian Common Buzzard (*Buteo buteo*) (n=3)				3																		1
Little Owl (*Athene noctua*) (n=5)				2				1			1								1			2
Goshawk (*Accipiter gentilis*) (n=1)				1																1		1
Tawny Owl (*Strix aluco*) (n=9)				6													1		1			1
Cormorant (*Phalacrocorax carbo*) (n=2)				2	1																	
Common Kestrel (*Falco tinnunculus*) (n=5)				3		1															1	1
Eurasian Hobby (*Falco subbuteo*) (n=1)					1																	

Eurasian Scops-owl (*Otus scops*) (n=2)	1											1	1					1			
Owl (*Asio otus*) (n=3)			2																		1
Barn Owl (*Tyto alba*) (n=3)			1			1															1
Eurasian Sparrowhawk (*Accipiter nisus*) (n=1)			1																		
Mallard (*Anas platyrhynchos*) (n=1)				1							1										
Great Crested Grebe (*Podiceps cristatus*) (n=1)			1																		
Swan (*Cignus olor*) (n=1)			1																		
Night Heron (*Nycticorax nycticorax*) (n=1)			1										1								
Common Pigeon (*Columba livia*) (n=3)			3																		
Eurasian Collared-dove (*Streptopelia decaocto*) (n=3)		1	1								1										1
Green Woodpecker (*Picus viridis*) (n=1)																					
Eurasian Hoopoe (*Upupa epops*) (n=1)			1																		
Common Redstart (*Phoenicurus phoenicurus*) (n=1)			1																		
Hooded Crow (*Corvus cornix*) (n=2)			2																		
Eurasian Magpie (*Pica pica*) (n=3)			3																		
Short-toed Snake Eagle (*Circaetus gallicus*) (n=3)			1								1										1
Common Gull (*Larus canus*) (n=1)			1																		
European Herring Gull (*Larus argentatus*)(n=1)			1																		
Eurasian Jay (*Garrulus glandarius*) (n=1)			1																		

Pheasant (*Phasianus colchicus*) (n=1)	1			1
Cattle Egret (*Bubulcus ibis*) (n=1)		1		
European Greenfinch (*Chloris chloris*) (n=1)			1	
Eurasian Siskin (*Spinus spinus*) (n=1)	1			

Table S3. Sources of MIC breakpoints used for interpreting AST results across different bacterial species.

Bacteria species	Method	Antimicrobial	Breakpoints source	References
Enterobacter spp., *K. pneumoniae* (Breakpoints defined for *Enterobacterales* in CLSI and EUCAST guidelines, and for *Enterobacteriaceae* in CA-SFM recommendations)	MIC by broth microdilution	AN	CA-SFM recommandations v1.0, 2014<br>Parolini et al.	[1,2]
		KAN, SFX, TET, AMC§, CFZ§	CLSI M100 35th ed., 2025	[3]
		CTX, CL, GEN, SXT, ENR*	EUCAST breakpoint tables for interpretation of MICs and zone diameters v15.0, 2025	[4]
		FFC	EUCAST ECOFFs (*E. coli; K. pneumoniae*)	[5]
		FLU	EUCAST ECOFFs (*E. coli*)	[5]
A. baumannii (Breakpoints defined for *Acinetobacter* spp.)	MIC by broth microdilution	CL, SXT, GEN	EUCAST breakpoint tables for interpretation of MICs and zone diameters v15.0, 2025	[4]
		ENR*	CLSI M100 35th ed., 2025	[3]
P. aeruginosa (Breakpoints defined for Pseudomonas spp. in EUCAST guidelines, and for *Pseudomonas aeruginosa* in CLSI guidelines)	MIC by broth microdilution	CL	EUCAST breakpoint tables for interpretation of MICs and zone diameters v15.0, 2025	[4]
		ENR*	CLSI M100 35th ed., 2025	[3]
		FFC	EUCAST ECOFFs (*P. aeruginosa*)	[5]
		GEN	CLSI M100 31st ed., 2021	[6]
E. faecium (Breakpoints defined for *Enterococcus* spp.)	MIC by broth microdilution	AMP	EUCAST breakpoint tables for interpretation of MICs and zone diameters v15.0, 2025	[4]
		AMC**	EUCAST breakpoint tables for interpretation of MICs and zone diameters v14.0, 2024	[7]
		ENR*, ERY, P, RD, TET	CLSI M100 35th ed., 2025	[3]
		FFC	EUCAST ECOFFs (*E. faecium*)	[5]

S. aureus<br><br>(Breakpoints defined for *Staphylococcus* spp.)	MIC by broth microdilution	P, OX, ENR*, SFX	CLSI M100 35th ed., 2025	[3]
		AMC[a], AMP[a], CFZ[a], CTF, FFC	EUCAST ECOFFs (*S. aureus*) [a]	[5]
		DA, ERY, RD, TET, SXT	EUCAST breakpoint tables for interpretation of MICs and zone diameters v15.0, 2025	[4]
		KAN	CA-SFM recommandations v1.1, 2025	[8]

§ Breakpoints were applied exclusively for the interpretation of MIC values obtained for *K. pneumoniae*, given the intrinsic resistance of *Enterobacter* spp. to these antimicrobials; *Breakpoints defined for ciprofloxacin MIC values interpretation were used for ENR; ** For the interpretation of MIC values defined for AMC, breakpoints provided by EUCAST v.14 (2024) guidelines were used as a reference, assuming nonetheless that EUCAST states that susceptibility for AMC can be inferred from AMP [4]; [a] For the interpretation of MIC values defined for AMP, AMC, and CFZ, the ECOFFs provided by EUCAST for *S. aureus* were used as a reference, assuming nonetheless that CLSI states that staphylococci susceptible to P and OX can be considered susceptible to other beta-lactam/beta-lactam combination agents with established clinical efficacy for staphylococcal infections (including AMP and AMC), as well as to cephems including CFZ [3]. AN: aminosidine; AMC: amoxicillin/clavulanic acid; AMP: Ampicillin; CFZ: cefazolin; CTX: cefotaxime; CL: colistin; ENR: enrofloxacin; FFC: florfenicol; FLU: flumequine; GEN: gentamicin; KAN: kanamycin; SFX: sulfisoxazole; TET: tetracycline; SXT: trimethoprim/sulfamethoxazole; CTF: ceftiofur; DA: clindamycin; ERY: erythromycin; OX: oxacillin; P: penicillin; RD: rifampicin; TIL: tilmicosin. CA-SFM: Comité de l'antibiogramme de la Société Française de Microbiologie; CLSI: Clinical and Laboratory Standards Institute; EUCAST: European Committee on Antimicrobial Susceptibility Testing.

References

1. CA-SFM/EUCAST. Comité de L'antibiogramme de la Societé Francaise de Microbiologie; Recommendations v.1.0. Available online: https://www.departement-information-medicale.com/wp-content/uploads/2015/04/CASFM_EUCAST_V1_0_2014.pdf (accessed on 20 April 2025).
2. Parolini, F.; Ventura, G.; Rosignoli, C.; Rota Nodari, S.; D'incau, M.; Marocchi, L.; Santucci, G.; Boldini, M.; Gradassi, M. Detection and phenotypic antimicrobial susceptibility of *Salmonella* enterica serotypes in dairy cattle farms in the Po valley, northern Italy. *Animals* **2024**, *14*, 2043, doi:https://doi.org/10.3390/ani14142043.
3. CLSI. *Performance Standards for Antimicrobial Susceptibility Testing*, 35th ed.; Clinical and Laboratory Standards Institute: Wayne, PA, USA, 2025; Volume M100.
4. EUCAST. Breakpoint tables for interpretation of MICs and zone diameters; Version 15.0. Available online: https://www.eucast.org/fileadmin/src/media/PDFs/EUCAST_files/Breakpoint_tables/v_15.0_Breakpoint_Tables.pdf (accessed on 14 April 2025).
5. EUCAST. Antimicrobial wild type distributions of microorganisms. Available online: https://mic.eucast.org/search/?search%5Bmethod%5D=mic&search%5Bantibiotic%5D=-1&search%5Bspecies%5D=-1&search%5Bdisk_content%5D=-1&search%5Blimit%5D=50 (accessed on 3 May 2024).
6. CLSI. *Performance Standards for Antimicrobial Susceptibility Testing*, 31st ed.; Clinical and Laboratory Standards Institute: Wayne, PA, USA, 2021; Volume M100.
7. EUCAST. Breakpoint tables for interpretation of MICs and zone diameters; Version 14.0. Available online: https://www.eucast.org/fileadmin/src/media/PDFs/EUCAST_files/Breakpoint_tables/v_14.0_Breakpoint_Tables.pdf (accessed on 14 April 2025).
8. CA-SFM/EUCAST. Comité de L'antibiogramme de la Societé Francaise de Microbiologie; Recommendations v.1.1. Available online: https://www.sfm-microbiologie.org/wp-content/uploads/2025/07/CASFM2025_V1.1-JUILLET-2025.pdf (accessed on 25 April 2025).
